# Combined Effect of Conventional Chemotherapy with Epigenetic Modulators on Glioblastoma

**DOI:** 10.3390/genes16020138

**Published:** 2025-01-24

**Authors:** Adrian Albulescu, Anca Botezatu, Alina Fudulu, Camelia Mia Hotnog, Marinela Bostan, Mirela Mihăilă, Iulia Virginia Iancu, Adriana Plesa, Lorelei Brasoveanu

**Affiliations:** 1 Molecular Virology Department, Stefan S. Nicolau Institute of Virology, Romanian Academy, 030304 Bucharest, Romania; adrian.albulescu@virology.ro (A.A.); alina.fudulu@virology.ro (A.F.); iulia.iancu@virology.ro (I.V.I.); adriana.plesa@virology.ro (A.P.); 2 Pharmacology Department, National Institute for Chemical Pharmaceutical Research and Development, 031299 Bucharest, Romania; 3 Center of Immunology, Stefan S. Nicolau Institute of Virology, Romanian Academy, 030304 Bucharest, Romania; camelia.hotnog@virology.ro (C.M.H.); marinela.bostan@virology.ro (M.B.); mirela.mihaila@virology.ro (M.M.); lorelei.brasoveanu@virology.ro (L.B.)

**Keywords:** lncRNA, glioblastoma, epigenetics, combined therapy, methylation, HDACi, PN 23 28 05 01ionsmonoclonal antibody

## Abstract

Background/Objectives: Glioblastoma is the most common malignant primary brain tumor, characterized by necrosis, uncontrolled proliferation, infiltration, angiogenesis, apoptosis resistance, and genomic instability. Epigenetic modifiers hold promise as adjuvant therapies for gliomas, with synergistic combinations being explored to enhance efficacy and reduce toxicity. This study aimed to evaluate the effects of single or combined treatments with various anticancer drugs (Carboplatin, Paclitaxel, Avastin), natural compounds (Quercetin), and epigenetic modulators (suberoylanilide hydroxamic acid and 5-Azacytidine) on the expression of some long noncoding RNAs and methylation drivers or some functional features in the U87-MG cell line. Methods: Treated and untreated U87-MG cells were used for the evaluation of drug-induced cytotoxicity, apoptotic events, and distribution in cell cycle phases, detection of cytokine release, and assessment of gene expression and global methylation. Results: Cytotoxicity assays led to the selection of drug concentrations to be used in further experiments. Expression analysis revealed distinct downregulation of nearly all investigated genes and long noncoding RNAs following treatments. All treatments resulted in a higher percentage of global methylation compared to untreated controls. All treatments effectively increased levels of apoptosis, while the epigenetic modulators exhibited a lower proliferation profile, with combined treatments showing elevated values of cell lysis. Conclusions: The results indicate a link between Carboplatin and Avastin treatments and DNA methylation mechanisms involving EZH2, DNMT3A, and DNMT3B, with Avastin’s direct impact on these enzymes warranting further study. This research underscores the promise of platinum-based therapies combined with epigenetic drugs to reactivate silenced tumor suppressor genes and optimize methylation profiles.

## 1. Introduction

Glioblastoma is the most common and aggressive form of tumor pathology of the central nervous system (CNS). The major difficulties posed by this pathology are given by the extremely high resistance of the tumors to conventional therapies and their invasive nature [1]. Statistics show that globally, approximately 320,000 cases of CNS cancer are diagnosed annually, of which almost half are glioblastomas [2]. Although cancer survival rates have risen steadily, the five-year survival rate for glioblastoma has shown little or no significant improvement over the past decades, varying from 3–5% to 7–10% in some cases [3,4,5,6,7,8,9,10]. The standard treatment plan for glioblastoma patients consists of surgical resection followed by chemotherapy with temozolomide (TMZ) and radiotherapy. However, the tumors almost invariably recur and challenges such as incomplete resection, high genetic heterogeneity, immunosuppressive environment, and the blood–brain barrier remain major obstacles to an effective treatment [11].

Despite recent advances in cancer therapy, resistance to chemotherapy, radiotherapy, targeted therapy, and immunotherapy remains extremely common in glioblastoma [12]. The recurrence rate is notably high, due to the infiltrative nature of the tumor cells which, often, cannot be completely removed through surgery. This form of cancer is characterized by distinctive features including a predisposition to necrosis, uncontrolled cell proliferation, diffuse infiltration, abundant angiogenesis, intense resistance to apoptosis, and aggressive genomic instability [13]. In this context, there is an urgent need to identify new therapeutic targets and approaches to combat this type of cancer which has an exceptionally high mortality rate [14].

To address the heterogeneity of this pathology, biological subclassification systems have been developed. The most prominent system relies on the presence of mutations in isocitrate dehydrogenase 1 (*IDH1*) or 2 (*IDH2*). However, the majority of primary glioblastomas lack IDH mutations [15,16].

The study of epigenetic modifications represents another piece of the glioblastoma puzzle, and recent experiments have shown differences in DNA methylation patterns among subgroups of glioblastoma patients [17,18,19,20,21].

Moreover, it has been demonstrated that the majority of RNA molecules present in cells do not actually encode proteins, and that noncoding species such as microRNA, long noncoding RNA (lncRNA), or circular RNA predominate over their protein-coding counterparts [22,23]. These RNA species are involved in distinctive cellular processes of cancer, such as proliferation, resistance to apoptosis, invasion, metastasis, and genomic instability. The aberrant expression profiles of lncRNA species correlate with the process of malignant transformation and have an important role in the classification, diagnosis, and prognosis of gliomas [24,25]. Moreover, lncRNAs are expressed in a cell-type-specific manner compared to transcription factors or other protein-coding genes and may constitute advantageous targets for future therapies based on personalized medicine [26].

Classical cancer drug treatment often involves cytostatics, such as Carboplatin (CPt) and 5-fluorouracil (5-FU), which inhibit DNA synthesis and disrupt cancer cell proliferation [27]. Taxanes like Paclitaxel (Pxl) are widely used for their ability to stabilize microtubules and block cell division, leading to apoptosis [28]. Complementary therapies, such as immunotherapy with monoclonal antibodies, offer targeted approaches to combat tumor progression. For instance, Avastin/Bevacizumab (Ava), an anti-angiogenic monoclonal antibody, inhibits vascular endothelial growth factor (*VEGF*), reducing tumor blood supply and growth [29]. Additionally, biologically active compounds derived from plants, such as Quercetin (Qct), have gained attention for their anticancer properties, including antioxidant, anti-inflammatory, and apoptosis-inducing effects, making them promising candidates for integrative therapeutic strategies [30].

In our group’s previous studies, we observed that several lncRNAs have a deregulated expression in U87-MG glioblastoma and SK-N-SH neuroblastoma cell lines compared to normal human astrocytes. We identified 26 overexpressed and 69 underexpressed lncRNA species in U87MG cells, first normalized against reference gene *U6* and double-normalized against normal human astrocytes (Lonza™, Antwerp, Belgium) (Supplementary Figure S1) [31]. Furthermore, from this pool, those with significantly altered expression profiles, limited data related to glioblastomas, or potential relevance as biomarkers in cancer pathologies were selected for further investigation and evaluated in a dozen patient samples [32]. The present article is a revised and expanded version of a conference report entitled “Long noncoding RNA profile in neuroblastoma and glioblastoma”, which was presented at “46th FEB Congress held with 25th IUBMB Congress and 15th PABMB Congress as ‘The Biochemistry Global Summit’, Lisbon, Portugal, 9–14 July 2022, P-01.1-042 according to https://febs.onlinelibrary.wiley.com/doi/epdf/10.1002/2211-5463.13440, accessed on 31 December 2024” [31].

In this context, we selected some of the identified lncRNAs (NRON, EMX2OS, ZFAS1, HAR1B, TUG1, MALAT1, H19, MEG3, HOTTIP, GAS5, HOTAIR) in order to assess the effect of single versus combined treatments of several anticancer drugs (Carboplatin/CPt, Paclitaxel/Pxl), monoclonal antibodies (Avastin/Ava/Bevacizumab), or natural compounds (Quercetin/Qct) with established epigenetic modulators like suberoylanilide hydroxamic acid (SAHA), a histone deacetylase inhibitor (HDACi), or 5-Azacytidine (5-Aza C), a demethylating agent, on the U87-MG glioblastoma cell line. In addition to the effect on lncRNA expression, we investigated a few epigenetic modulators (EZH2, DNMT1, DNMT3A, and DNMT3B) and the drug-induced modulation of the cell proliferation profile or the impact on some biological activities (apoptosis, cell cycle distribution, cytokine release).

## 2. Materials and Methods

### 2.1. Reagents

Suberoylanilide hydroxamic acid (SAHA) and 5-Aza-Citidine (5-Aza C) were purchased from Sigma Aldrich (St. Louis, MO, USA); Avastin (Ava) was purchased as a 25 mg/mL concentrate for infusion of Bevacizumab (Roche Reg. Ltd., Welwyn Garden City, UK); Carboplatin (CPt) was obtained as a 10 mg/mL concentrate for infusion from Teva Ltd. UK (Eastbourne, East Sussex, UK), while Paclitaxel (Pxl) was purchased as a 6 mg/mL concentrate for infusion from Fresenius Kabi AG (Bad Homburg, Germany). Quercetin (Qct), ethylenediaminetetraacetic acid (EDTA), dimethyl sulfoxide (DMSO), and paraformaldehyde (PFA) were obtained from Sigma Aldrich (St. Louis, MO, USA).

The human IL-6 and TNF-α uncoated ELISA kits were obtained from Invitrogen (Bender MedSystems GmbH, Vienna, Austria). The Annexin V-FITC Apoptosis Detection kit was purchased from Becton Dickinson (BD) Biosciences (Mountain View, CA, USA). The FxCycle PI/RNase Staining solution was from Invitrogen by Thermo Fisher Scientific (Life Technologies Corporation, Eugene, OR, USA).

### 2.2. Cell Culture and Treatments

The U87-MG cell line was obtained from the American Type Culture Collection (ATCC^®^ HTB-14™, Manassas, VA, USA) and was first isolated from a male patient’s glioma. The cells were routinely maintained in culture according to the provider’s instructions, in an EMEM medium supplemented with 10% Fetal Bovine Serum, at 37 °C and in a 5% CO_2_ humidified atmosphere. When the cells grown in culture flasks of 25 or 75 cm^2^ achieved around 60–70% confluence, they were treated with various concentrations of epigenetic modulators (SAHA, 5-Aza C) and/or oncolytic drugs (CPt, Pxl), monoclonal antibodies (Ava), or natural compounds (Qct) for 24–48h. Then, the cells from the flasks were detached with a non-enzymatic solution of PBS/1mM EDTA, washed twice in PBS and immediately used for drug-cytotoxicity assays or the evaluation of apoptosis by flow cytometry technique or preserved for the extraction of nucleic acids for the molecular biology assays. Alternatively, when cells were used for cell cycle analysis by flow cytometry, they were fixed in ice-cold ethanol/PBS (70:30) and kept until use at 4 °C. In addition, cell culture supernatants were taken, centrifuged at 400× *g* and preserved at −80 °C until use for the evaluation of soluble cytokines. Non-treated (NT) cells were used as controls throughout all experiments [33,34].

### 2.3. Cytotoxicity Assays and Cell Viability (MTS)

The capacity of several epigenetic drugs (SAHA), oncolytic drugs (CPpt, Pxl), or other biocompounds (Qct) to inhibit cell proliferation was investigated in the U87-MG glioma cell line in order to modulate the chemosensitivity of glioma cells to drug treatments, thus overcoming or reversing the chemo-resistance. The cytotoxic potential of SAHA, CPt, Pxl, and Qct was assessed using the MTS assay, a colorimetric cell viability method [35]. All experiments were performed in triplicate in 96-well microtiter plates with a flat bottom (Falcon), using the CellTiter 96^®^ AQueous One Solution Cell Proliferation Assay kit (Promega, Madison, WI, USA). This assay employs a reagent mixture containing MTS [3-(4,5-dimethylthiazol-2-yl)-5-(3-carboxymethoxyphenyl)-2-(4-sulfophenyl)-2H-tetrazolium], a tetrazolium compound, and PES (phenazine ethosulfate), a cationic dye with high chemical stability that forms a stable solution with MTS [36].

The assay principle is based on the ability of metabolically active cells to reduce MTS, a yellow tetrazolium salt, to formazan, a soluble colored product in the culture medium that is spectrophotometrically measurable at a wavelength of 492 nm. Briefly, 10^4^ cells/well were cultured in 100 µL in 96-well flat-bottom plates for 24 h, and then the culture supernatants were discarded, and cells were treated for additional 24–48 h with 6 increasing concentrations of drugs: CPt, SAHA, and Qct (6.25, 12.5, 25, 50, 100, and 200 µM each), or Pxl (0.125, 0.25, 0.5, 1, 2, and 4 µM).

Following treatment, 20 µL of the MTS reagent mixture was added to each well, and the plates were incubated at 37 °C for 4 h, with gentle shaking every 20 min. The color developed during incubation was spectrophotometrically quantified, the absorbance was measured at λ = 492 nm using a Dynex ELISA reader (DYNEX Technologies—MRS, Chantilly, VA, USA) [37].

Cell viability was expressed relative to untreated cells (NT) as percentage, where control cells were considered to have 100% viability, and calculated using the following formula:

Cell viability (%) = 100 × (T − B)/(U − B),

where T represents the optical density of treated cells, B is the optical density of the blank (culture medium without cells), and U is the optical density of untreated cells.

The results were reported as mean values ± standard deviation (SD) from three independent experiments (*n* = 3) [38]. The cytotoxicity of DMSO was also assessed under identical experimental conditions, and no reduction in cell viability was observed at concentrations below 1%. Additionally, to account for the potential nonspecific reactions between MTS and the tested substances, including SAHA, CPt, Paclitaxel, and Quercetin, their absorbance was measured in the absence of cells, and these values were subtracted during data analysis.

### 2.4. Cell Cycle Analysis by Flow Cytometry

To reveal the effects of epigenetic drugs, alone or combined with other drugs, that may have a modulatory potential on the distribution of U87-MG cell populations in various phases of the cell cycle, we analyzed the DNA content of the cells by flow cytometry approaches. Modifications of DNA progression through cell cycle phases may account for the anti-carcinogenic effects of various drugs. Briefly, 10^6^ non-treated (NT) control or drug-treated U87-MG glioma cells with 5 µM of SAHA or 5-Aza C epigenetic drugs, used either alone or combined with oncolytic drugs (50 µM of CPt or 1 µM Pxl), monoclonal antibodies (20 µg/mL of Ava), or natural compounds (50 µM of Qct), fixed and preserved as described above, were twice washed with cold PBS and centrifuged for 5 min at 300× *g* at 4 °C. Then, cells were further subjected to RNase treatment and propidium iodide (PI) labeling, by using FxCycle™PI/RNase Staining Solution (Invitrogen, Waltham, MA, USA), a PI solution that includes DNase-free RNase A and a permeabilization reagent. After the buffer was removed from the cell pellet, 0.5 mL of the FxCycle™PI/RNase Solution was added, the cell pellet was gently vortexed, and resuspended cells were further incubated for 10–30 min at room temperature (RT) in the dark. Data acquisition of 3 × 10^4^ events was performed by flow cytometry, using a FACS CANTO II flow cytometer (BD Biosciences, Franklin Lakes, NJ, USA) and DIVA 6.2 software. The cell cycle analyses were performed using ModFIT LT software to estimate the nuclear DNA content in nuclei and the progression through cell cycle phases [39].

### 2.5. Apoptosis Analysis by Flow Cytometry

In order to study the potential role of epigenetic drugs in the induction or modulation of apoptosis in glioma cancer cells, the levels of apoptotic events were assessed by flow cytometry techniques. The U87-MG glioma cells were cultured in complete medium for 24 h, and then the culture medium was changed, and cells were treated for an additional 48 h with 5 μM of SAHA or 5-Aza C epigenetic drugs, used either alone or combined with 50 μM of CPt or 1 μM Pxl, 20 μg/mL of Ava or 50 μM of Qct. Then, U87-MG cells were detached with PBS/1 mM EDTA, sequentially washed with PBS, and centrifuged for 5 min at 300× *g*. Then, cell pellets were suspended in 400 μL of binding buffer, distributed in flow tubes (100 µL/tube) and stained with 5 µL of Annexin-V/FITC and/or PI at RT for 15 min in the dark). The negative control was represented by untreated (NT) cells. The green and red fluorescences were measured by using a FACS CANTO II flow cytometer (BD Biosciences, Immunocytometry System, Mountain View, CA, USA) after acquiring 10,000 events. Then, the acquired data were analyzed using DIVA 6.2 software, and apoptotic events were expressed as cell percentages.

### 2.6. Evaluation of Cytokine Levels by ELISA

Cytokines are peptides and proteins primarily secreted by activated immune cells, serving as key mediators of communication within the immune system. Their primary role is to regulate and mediate immune and inflammatory responses. Key cytokines such as TNF-α, IL-1, IL-6, and IL-12 play crucial roles in macrophage phagocytosis, antigen presentation, and inflammation regulation. Monitoring the activity of cytokines is essential due to their widespread impact on biological processes, which can be either beneficial or harmful depending on the levels produced and the surrounding conditions [40,41].

To assess the levels of several released cytokines by drug-treated glioma cells vs. untreated cells in conditioned media (cell culture supernatants), Enzyme-linked Immunosorbent Assays (ELISAs) for the quantitative detection of assays were performed. Thus, Human IL-6 Uncoated ELISA and Human TNF-α Uncoated ELISA kits (Invitrogen, Bender MedSystems GmbH, Vienna, Austria) were used, and the method was based on the measurement of the attached proteins of interest to biotin-conjugated specific antibodies coupled with the streptavidin–HRP complex enzyme. Briefly, 96-well plates were coated with specific capture antibodies and incubated overnight at 4 °C. After washing with 0.05% PBS-Tween 20 buffer, 100 µL of standard dilutions and conditioned media were added and incubated at 4 °C. After two other washings with PBS-Tween 20 buffer, the detection specific antibodies were added and plates were further incubated for 1h at RT. Then, the streptavidin–HRP complex was then added for 30 min at RT, followed by the TMB substrate solution for 15 min at room temperature. The reaction was stopped by (NH_4_)_2_SO_4_ solution, and optical densities were spectrophotometrically measured at λ = 450 nm. The standard curves were designed by eight 1:2 serial dilutions (concentration points), starting from 500 pg/mL for IL-6 and 1000 pg/mL for TNF-α. The standard curves were linearly calculated according to the kit recommendations. The assay values for each cytokine were obtained from the standard curves and expressed in pg/mL. After calculations, the results for the treatment-induced cytokine release were normalized by dividing the values by the untreated cell concentration, considered 100%.

### 2.7. RNA Isolation and cDNA Synthesis

Total RNA extraction and purification were carried out for all samples using the RNeasy Mini kit (Qiagen, Hilden, Germany), according to the manufacturer’s protocol. The isolated RNA was then reverse-transcribed into cDNA using the High-Capacity cDNA Reverse Transcription Kit (Thermo Fisher Scientific Inc., Waltham, MA, USA), with 1 µg of RNA from each sample as input.

### 2.8. Quantitative Analysis of Selected Genes and lncRNA

mRNA expression levels were detected using qRT-PCR on an Applied Biosystems 7300 Real-Time PCR system (Applied Biosystems, Foster City, CA, USA), with U6 serving as the reference gene. The qRT-PCR reactions were carried out in a total volume of 25 µL, consisting of 12.5 µL of Maxima SYBR Green/ROX qPCR Master Mix (2×) (Thermo Fisher Scientific, Waltham, MA, USA), 0.30 µM of each primer, and 50 ng of target cDNA. All experiments were performed in triplicate, and relative expression was calculated using the 2^−∆Cq^/2^−ΔΔCq^ method, and the log10 values used for further visualization and analysis of the data. The primer list with specific sequences for selected genes is available in Supplementary Table S1. Most of the primer pairs were custom-made for this study, while a few (U6, H19, and EZH2) were based on available literature data [37,42,43,44].

### 2.9. DNA Isolation and LINE-1 Global Methylation Assay

DNA isolation from treated and untreated cells was performed using the QIAamp DNA Mini Kit (Qiagen, Hilden, Germany) according to the producer’s guidelines. The concentration and purity of each DNA sample were assessed using a NanoDrop ND-1000 spectrophotometer (Thermo Fisher Scientific Inc., Waltham, MA, USA).

LINE-1 methylation was quantified using the Global DNA Methylation LINE-1 Kit (Active Motif, Carlsbad, CA, USA), an ELISA-based assay for detecting 5-methylcytosine (5-mC) levels in genomic DNA. Briefly, genomic DNA was extracted, digested with the MseI enzyme (10 U/μL) at 37 °C overnight, and hybridized with a biotinylated LINE-1 probe targeting a CpG-rich region of the LINE-1 repeat element. The hybridized treated sample DNA and standard controls were loaded in triplicate onto streptavidin-coated plates and sequentially incubated with a 5-mC-specific antibody and an HRP-conjugated secondary antibody. A colorimetric reaction was then initiated using Developing Solution, and stopped by adding Stop Solution as soon as the highest concentration DNA standards displayed a medium blue color. The absorbance was measured at 450 nm with a reference wavelength of 655 nm, using a model microplate reader (TriStar^2^S, Berthold Technologies, Bad Wildbad, Germany). The results were expressed as the percentage of 5-mC relative to total cytosine content, with standards processed in parallel.

### 2.10. Statistical Analysis

Statistical analyses were performed using GraphPad Prism version 10.3 (GraphPad Software Inc., San Diego, CA, USA) on triplicate samples, with *p*-values < 0.05 considered statistically significant. For gene and lncRNA experiments, data were expressed as log_10_ medians and analyzed using the Mann–Whitney nonparametric test and Student *t*-test when appropiate. For correlation studies, Pearson’s test was employed on n-fold target gene expression datasets. For cytotoxicity tests and ELISA assays, the results were reported as mean values ± standard deviation (SD) and statistically analyzed using the Student *t*-test and one-way ANOVA.

## 3. Results

### 3.1. Drug-Induced Cell Cytotoxicity Is Dose- and Time-Dependent

**Figure 1:**
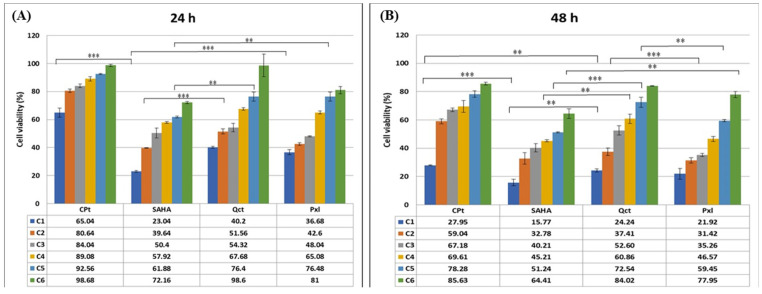
Drug-cytotoxicity effects on U87-MG glioma cells due to six different concentrations each of Cpt, SAHA, Qct, and Pxl. Panel (**A**) 24 h; Panel (**B**) 48 h. (** *p* < 0.005, *** *p* < 0.0005).

The capacity of several epigenetic drugs (SAHA), oncolytic drugs (CPt, Pxl), or other biocompounds (Qct) to inhibit cell proliferation was investigated in the U87-MG glioma cell line in order to modulate the chemosensitivity of glioma cells to drug treatments, thus overcoming or reversing the chemo-resistance. The percentages of cell viability were decreased by drug treatments in the U87-MG cell line as shown in Figure 1, panels A and B.

In an analysis of the cell response to treatments, the strongest cytotoxic effects were observed when glioma cells were treated for 48 h. The cell response to drug treatments demonstrated a cytotoxic dose- and time-dependent effect for all tested drugs, inducing an increase in cell lysis rates, and implicitly decreased cell viability. The cytotoxic profiles of the various drugs used throughout our study are shown in Figure 1. Thus, treatments for 24 h with 200 µM of SAHA or Qct decreased the U87-MG cell viability to 23% and 40.2%, respectively; the 100 µM and 50 µM treatments with the same drugs decreased the viability to 39.64% and 50.4% for SAHA, and to 51.56 and 54.32% for Qct. A strong effect was also shown for smaller concentrations of Pxl since the 4, 2, and 1 μM concentrations decreased the cell viability to 36.68%, 42.6%, and 48.04%, respectively (Figure 1A). Lower effects were observed for CPt, with the concentration of 200 µM being able to induce a decrease in cell viability to 65% (Figure 1A). The effect of drugs on the cell viability of U87-MG cells highlights the impact of concentration: the prolonged treatments for 48 h induced higher decreases in the viability percentages as compared to the results obtained after 24 h of treatment (Figure 1B). Thus, concentrations of SAHA between 25 and 200 µM decreased the cell viabilities between 15.8% and 45.21%. The same stronger effects were observed for Qct and Pxl, but also 200 µM of CPt induced a decrease in the cell viability to 28% (Figure 1B).

Cell cytotoxicity was induced by 24 h or 48 h treatments of U87-MG cells with six scalar concentrations each of SAHA (200, 100, 50, 25, 12.5, 6.25 µM), CPt (200, 100, 50, 25, 12.5, 6.25 µM), Pxl (4, 2, 1, 0.5, 0.25, 0.125 µM), and Qct (200, 100, 50, 25, 12.5, 6.25 µM) and compared to untreated control cells. Data shown are expressed as mean values ± standard deviations (SDs) of three different experiments (n = 3) and were statistically analyzed using the Student *t*-test and one-way ANOVA (** *p* < 0.005, *** *p* < 0.0005).

The obtained results after data analysis following the MTS colorimetric assay prompted us to use the cells subjected to 48h of treatments with fixed concentrations of 5 μM for SAHA, 50 μM for CPt, 1 μM for Pxl, and 50 μM for Qct in subsequent end-point assays, like apoptosis, DNA progression through cell cycle phases, and cytokine release, or during the molecular biology assays. Regarding 5-Aza C, the concentration was selected according to our previous results (C = 5 µM) [37]. Similarly, Ava has demonstrated its biological activity by binding VEGF at concentrations as low as 5 µg/mL, while the dosage used in patients is considerably higher. Based on this, the selected concentration was 20 µg/mL, as it effectively performs its biological function without exhibiting any cytotoxic effects [45].

### 3.2. Modulation of Cell Cycle Phases in Treated U87-MG Glioma Cells

**Figure 2:**
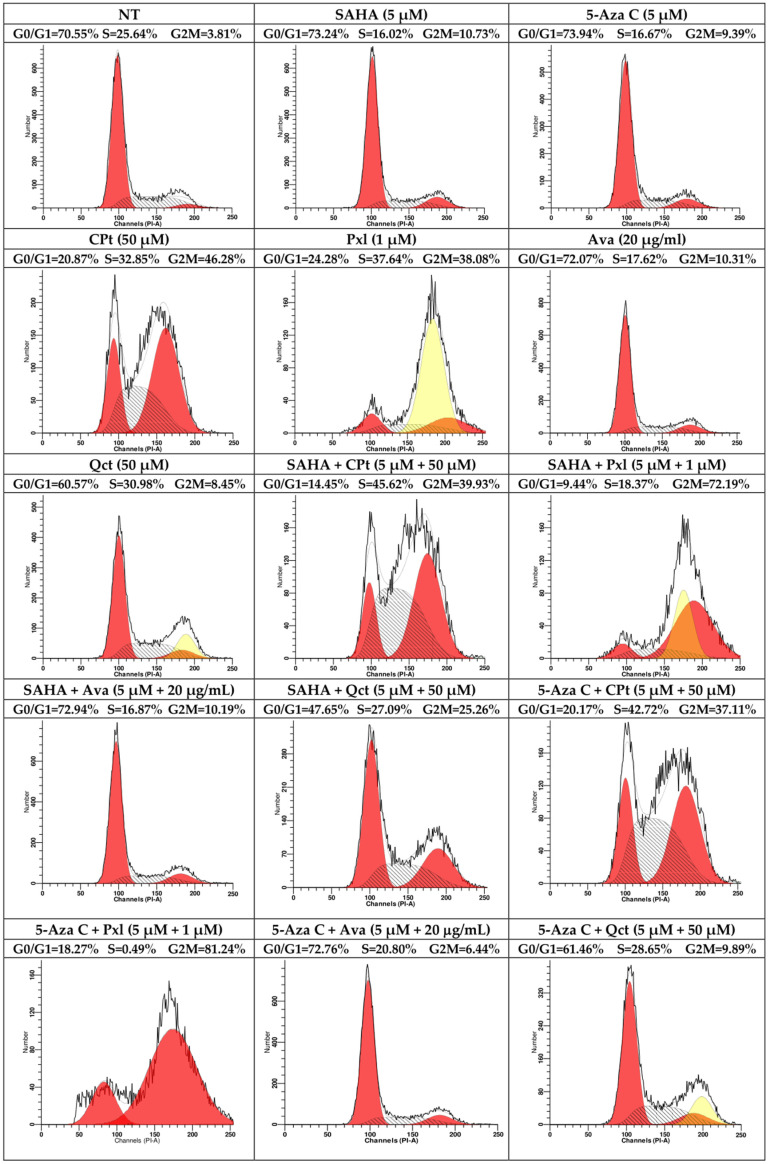
Modulation of U87-MG cell cycle progression by single vs. combined treatments. Red indicates G0/G1, respectively G2 phase, while the S phase is represented by shaded areas. Black outlines correspond to fluorescence intensity or the number of events, and yellow denotes doublets cells.

Control cells displayed a cell distribution of 70.55% in G0/G1, 25.64% in S, and 3.81% in G2M cell cycle phases. Single treatments with SAHA, 5-Aza C, or Ava induced a decrease in U87-MG cell proliferation, since cell distribution in the S phase diminished to 16.02%, 16.67%, and 17.62%, respectively; CPt and Pxl treatments induced a high decrease in the G0/G1 phase, concomitant with a high increase in the proliferation indices. The same effect of a decrease in percentages of nuclei in G0/G1 phase and increase in the proliferation indices was also observed for the combined treatments of SAHA or 5-Aza C with CPt and Pxl, where NT represents non-treated U87-MG cells (Figure 2, Supplementary Table S2).

### 3.3. Apoptosis Analysis in Treated U87-MG Glioma Cells

Annexin V, a calcium-dependent phospholipid-binding protein, binds to phosphatidylserine (PS) and thus allows the identification of apoptotic cells, either found in early (quadrant Q4) or late (quadrant Q2) stages, the last process preceding necrosis.

Effects of treatments with epigenetic drugs and other compounds on modulation of cell cycle phases were studied in the U87-MG glioma cell line and compared to untreated cells. The single treatments with Pxl, Ava, and Qct induced an increase in early apoptotic events, the highest effect being observed for Pxl, Ava, and Qct. The strongest effect of combined treatments was obtained for the combinations of SAHA with Pxl, CPt, or Qct, and also for 5-Aza C and CPt (Table 1) (Supplementary Figure S2).

### 3.4. Evaluation of Soluble Cytokines Released by Treated U87-MG Cells

**Figure 3:**
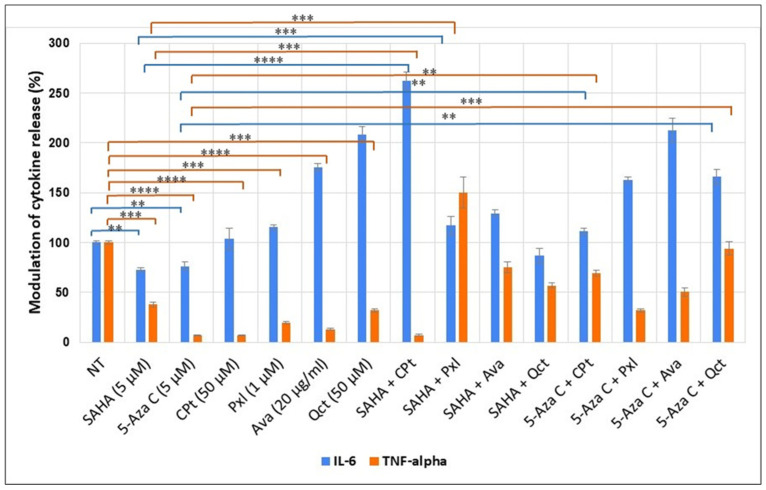
Modulation of IL-6 and TNF-α release by single or combined treatments in U87-MG cells. (** *p* < 0.005, *** *p* < 0.0005, **** *p* < 0.0001).

Supernatant cytokine normalization of treated against untreated cells, displayed in Figure 3, revealed strong pro-inflammatory responses in multiple treatment variants in the case of IL-6 (Figure 3).

The cytokine release was induced by 48 h single or combined treatments of U87-MG glioma cells with 5 µM of SAHA, 50 µM of CPt, 1 µM of Pxl, and 50 µM of Qct. The results for treatment-induced cytokine release were normalized by dividing the values by the non-treated (NT) cell concentration, considered 100%*,* and statistically analyzed using the Student *t*-test and one-way ANOVA (** *p* < 0.005, *** *p* < 0.0005, **** *p* < 0.0001).

While SAHA alone led to an anti-inflammatory reaction noted by a 27% decrease compared to untreated, and CPt shows a minimal inflammatory response (3.9% increase), the combined treatment led to a strong pro-inflammatory response marked by a 262% increase in the IL-6 relative abundance. In contrast, Qct alone provoked a sharp inflammatory response with a 208% raise, and Qct with 5-Aza C also led to an inflammatory response with a 163% increase in IL-6 relative levels, while in combination with SAHA, it led to a similar response in comparison to SAHA alone (87% relative abundance). Interestingly, TNF-α displayed all-around lower relative levels with the exception of SAHA + Pxl (150% increase), which had a pro-inflammatory response in the case of IL-6 as well (116% abundance). The higher TNF-α response of SAHA and Pxl is coupled with the highest proliferation index and also an increase in early and late apoptosis. Pxl with 5-Aza C did not elevate TNF-α levels as much as in combination with SAHA but also displayed a high number of cells in the G2 phase and the second-highest proliferation index. Ava maintained a strong response throughout all treatment variants concerning IL-6, while TNF-α relative levels increased from 13% in Ava-treated cells to 50% with 5-Aza C and up to 75% in combination with SAHA.

### 3.5. Target lncRNAs Gene Expression Quantification

In our previous data, we observed significantly increased expression levels of H19 (*p* = 0.0144), EZH2 (*p* = 0.0330), and HOTAIR in patients. By contrast, MALAT1 (*p* = 0.0025), MEG3, and GAS5 showed reduced expression levels. HOTTIP did not display any significant changes in expression levels [46].

In the present study, the gene expression of the selected lncRNAs and epigenetic modulators was evaluated regarding each treatment combination. Almost all lncRNAs showed a decreased expression under these treatments.

**Figure 4:**
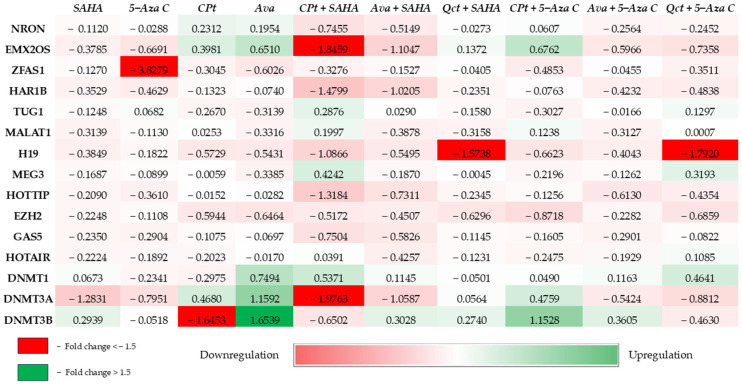
Heatmap of log_10_ values of double-normalized (relative to *U6* and relative to untreated cells) gene expression of selected lncRNAs and genes in experimental models. The color gradient represents expression levels, with red shades (downregulation), green shades (upregulation), and color intensity reflecting the magnitude of change.

The analysis also revealed that 5-Aza C and CPt alone inhibited the expression of *DNMT3B*, yet their combination induced a strong and statistically significant *DNMT3B* activation (Figure 4) (relative to NT *p* = 0.0014; relative to Cpt *p* = 0.0011; relative to 5-Aza C *p* < 0.0001, Supplementary Table S3). Ava also improved DNMT function with statistical significance (relative to NT, *DNMT1 p* < 0.0001; *DNMT3A p* = 0.0009; *p* < 0.0001).

The combination that modified the expression significantly compared with NT (control) for most of the selected genes was CPt + SAHA (nine genes), Ava and Ava + SAHA both (eight genes), and Qct + 5 Aza C (six genes).

*H19* expression was silenced by all treatments, showing somewhat less statistical significance in 5-Aza C, although in addition to Qct (Qct + 5-Aza C), it led to the sharpest decrease in expression (1.79 negative fold change, *p* = 0.0005 relative to NT). *H19* expression was also affected by Qct + SAHA treatment, presenting a 1.5-fold decrease relative to NT (*p* < 0.0001). *MALAT1* presented statistically significant modified gene expression in five treatment options. *MALAT1* gene expression was marginally upregulated in four combinations of treatment and slightly downregulated in six cases, most notable relative to NT when Ava + SAHA (*p* = 0.0244) was used. *EMX2OS* gene expression decreased in six treatments, reaching a 1.84-fold change relative to NT (*p* < 0.0001) in CPt + SAHA treatment. HAR1B (1.48 negative fold change, *p* = 0.0004 relative to NT) and HOTTIP (1.3 negative fold change, *p* = 0.0003 relative to NT) were also downregulated by CPt + SAHA. *ZFAS1*, *HAR1B*, and *HOTTIP* presented downregulated gene expression in all treatment combinations. For *ZFAS1*, 5-Aza C treatment had a notably powerful silencing effect (almost 4-fold decrease) with statistical significance (relative to NT *p* < 0.0001). *NRON* showed downregulated gene expression in most cases, with the sharpest silencing (0.74-fold change) induced by CPt + SAHA (*p* = 0.0009). *NRON* and *HAR1B* demonstrate a significantly decreased expression when comparing treatment combinations: CPt + SAHA relative to SAHA (*p* = 0.0006 and *p* = 0.0186, respectively), CPt + SAHA relative to CPt (*p* = 0.0171 and *p* = 0.0003, respectively), and Ava + SAHA relative to Ava (*p* = 0.0089 and *p* = 0.003, respectively). *EMX2OS* expression significantly decreases (1.46-fold change) when comparing CPt + SAHA relative to SAHA (*p* = 0.0002) and Cpt + SAHA relative to Cpt (2.24-fold change, *p* = 0.0001). Similarly, a significant decrease is observed for Ava + SAHA relative to Ava (1.75-fold change, *p* < 0.0001) and Ava + SAHA relative to SAHA (0.72-fold change, *p* = 0.0204). For *TUG1*, *MEG3*, *GAS5*, and *HOTAIR*, even though there are differences between each treatment, the gene expression level is not significantly modified (Figure 4, Supplementary Table S3).

**Figure 5:**
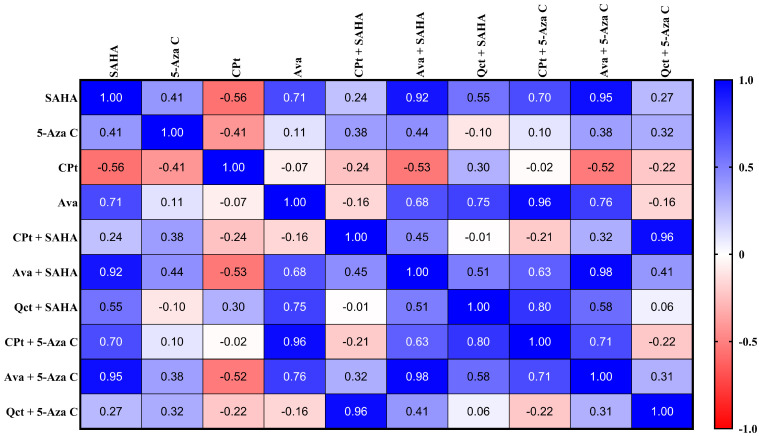
Correlogram showing the correlation coefficients of target gene expression profiles across different treatment conditions. Each cell represents the Pearson correlation coefficient between two treatments, with the color intensity and scale indicating the strength and direction of the correlation (positive in blue and negative in red).

The Pearson correlation analysis identified significant similarities in gene expression profiles across various treatments (Figure 5). SAHA treatment exhibited strong positive correlations with multiple treatments, including Ava + 5-Aza C (r^2^ = 0.95, *p* < 0.0001), Ava + SAHA (r^2^ = 0.92, *p* < 0.0001), Ava (r^2^ = 0.71, *p* = 0.0030), and CPt + 5-Aza C (r^2^ = 0.70, *p* = 0.0038). In contrast, SAHA showed a negative correlation with CPt (r^2^ = −0.56, *p* = 0.0315). Similarly, CPt demonstrated negative correlations with Ava + SAHA (r^2^ = −0.53, *p* = 0.0418) and Ava + 5-Aza C (r^2^ = −0.52, *p* = 0.0447).

Cells treated with Ava displayed similar gene expression profiles to those treated with CPt + 5-Aza C (r^2^ = 0.96, *p* < 0.0001), Ava + 5-Aza C (r^2^ = 0.76, *p* = 0.0009), and Qct + SAHA (r^2^ = 0.75, *p* = 0.0013). When SAHA was combined with other anticancer drugs, it did not consistently produce positive or negative correlations. However, CPt + SAHA showed a positive correlation only with Qct + 5-Aza C (r^2^ = 0.96, *p* < 0.0001). Ava + SAHA demonstrated a strong positive correlation with Ava + 5-Aza C (r^2^ = 0.98, *p* < 0.0001), while Qct + SAHA was positively correlated with CPt + 5-Aza C (r^2^ = 0.80, *p* = 0.0004). Additionally, a positive correlation was observed between CPt + 5-Aza C and Ava + 5-Aza C (r^2^ = 0.71, *p* = 0.0031). No significant correlations were found for gene expression in cells treated solely with 5-Aza C.

### 3.6. Epigenetic Modulators Exhibited Modified Expression Pattern and Global Methylation

All the epigenetic modulators investigated (*EZH2*, *DNMT1*, *DNMT3A*, and *DNMT3B*) displayed significant modification in gene expression depending on the treatment combinations. *EZH2* showed decreased levels of gene expression in almost all cases, the most notable being CPt + SAHA (*p* = 0.0041) and Ava (*p* = 0.0105). *DNMT1* presented increased gene expression in most of the treatments, with the sharpest upregulation in cells treated with Ava (*p* < 0.0001) or CPt + SAHA relative to NT (*p* = 0.0007), but a decrease in gene expression was observed when CPt treatment was used (*p* = 0.0178). *DNMT3A* expression was downregulated by the majority of treatment combinations, with CPt + SAHA (1.9-fold change, *p* < 0.0001) and SAHA single treatment (1.2-fold change, *p* = 0.0005) relative to NT being notable. Interestingly, the treatment with Ava determined a notable increase in gene expression (1.15-fold, *p* = 0.0009). *DNMT3B* gene expression is also modulated, being significantly decreased (1.64-fold change, *p* < 0.0001) in the case of CPt treatment and significantly increased by Ava (1.65-fold change, *p* < 0.0001) relative to NT. Another notable effect was observed in the case of CPt + 5-Aza C treatment, which determined an increase in *DNMT3B* expression (1.15-fold change, *p* = 0.0014) as opposed to the individual treatments. *DNMT1* expression increases when comparing CPt + SAHA to SAHA (*p* = 0.0015), CPt + SAHA to CPt (*p* < 0.0001), and CPt + 5-Aza C to CPt (*p* = 0.0085).

**Figure 6:**
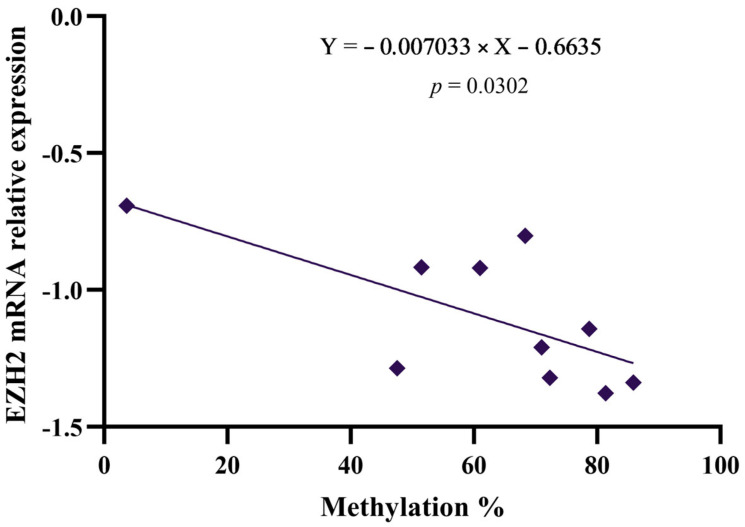
Correlation between global methylation and levels of *EZH2* gene expression in treated cells.

Global methylation results were expressed as mean percentage values and are presented in Supplementary Table S4. Further, a linear regression analysis between global methylation percentages and epigenetic factors *DNMT1*, *DNMT3A*, *DNMT3B*, and *EZH2* revealed a significant correlation in the case of *EZH2* (Figure 6).

## 4. Discussion

Apoptosis, or the programmed cell death mechanism, plays a vital role in maintaining tissue homeostasis by removing cells with DNA mutations, abnormal cycles or a high risk of malignant transformation [47]. However, during tumorigenesis, disruptions in apoptotic pathways result in unchecked cellular proliferation and resistance to therapies, like chemotherapy and radiotherapy [48]. Apoptosis serves as a critical target in cancer therapy, with anticancer drugs aiming to reactivate its pathways, and to induce selectively cancer cell death while preserving normal tissue, making it a key measure of therapeutic efficacy alongside assessments of cell proliferation inhibition [49].

The current study evaluates the apoptotic effects of various epigenetic modulators, cytostatics, and their combinations in glioblastoma U87-MG cells. Our findings demonstrate significant variability in apoptosis induction depending on the treatment, underscoring the potential of specific agents and their combinations for targeted therapy. However, there are several limitations in the present study. The experimental model relied on using only the U87MG glioblastoma cell line, and while it is a well-characterized model, it does not fully represent the heterogeneity of glioblastoma subtypes encountered in patients. Expanding these findings in genetically diverse cell lines or primary cultures would strengthen the significance of the results obtained. Another limitation of this study is due to the absence of any in vivo testing, which would be required in order to verify the efficacy and safety of these treatment combinations before clinical applications. Due to the use of specific concentrations for the test compounds and fixed time points, it is possible that alternative dose ranges or extended treatment durations could yield different epigenetic or cytotoxic effects. Employing a targeted approach that explores a defined panel of lncRNAs and epigenetic regulators may overlook other relevant molecular pathways or regulatory RNAs that also contribute to glioblastoma progression and therapy resistance, and thus, a broader transcriptomic or epigenetic profiling may reveal additional significant targets or mechanisms. Another limitation found in the present study is due to the lack of microenvironmental and immune components, which influence processes like angiogenesis, cell immune responses, and other stromal factors found in in vivo settings. Overcoming some of these limitations represents a future direction that could help shed some light on the complexities and intricacies of the mechanisms that drive glioblastoma resistance and aggressive behavior.

Among the single-agent treatments, Qct (50 μM) induced the highest total apoptosis (26.8%), significantly exceeding other treatments such as SAHA (12.7%) and 5-Aza C (13.6%). These results align with previous findings suggesting that plant-derived compounds like Quercetin exert strong anti-tumor effects by modulating apoptosis-related pathways [50]. Notably, Qct also exhibited a balanced effect on both early and late apoptosis phases, indicating its robust potential in disrupting tumor cell viability.

Combinatorial treatments provided valuable insights, with SAHA + Qct demonstrating one of the highest levels of total apoptosis (25.2%), comparable to Qct alone but with enhanced late apoptosis (16.5%). Similarly, SAHA + CPt induced a total apoptosis of 22.9%, emphasizing the potential of synergistic interactions between epigenetic modulators and cytostatics. In contrast, combinations involving 5-Aza C resulted in moderate apoptosis levels, with 5-Aza C + Qct showing the highest response (19.3%), highlighting how the selection of epigenetic modulators influences the effectiveness of combination therapies due to differences in their mechanisms of action. These findings suggest that the choice of epigenetic modulator significantly influences the efficacy of combination therapies, potentially linked to differences in their mechanisms of action.

Interestingly, while SAHA and 5-Aza C demonstrated moderate apoptotic effects as single agents, their efficacy was notably enhanced when combined with agents like Qct and CPt. This improvement highlights the therapeutic potential of pairing histone deacetylase inhibitors or DNA methyltransferase inhibitors with cytostatics or biologically active compounds to amplify apoptotic responses.

The strong apoptotic effect of Qct, likely due to its ability to disrupt mitochondrial function and oxidative stress, and the synergic interactions in combinations like SAHA + Cpt emphasize the need for further mechanistic studies to optimize these therapies.

Overall, the differential apoptotic responses observed in this study highlight the importance of selecting appropriate agents and combinations for glioblastoma therapy. The strong apoptotic potential of Qct, particularly in combination with SAHA, supports its inclusion in future preclinical and clinical investigations.

IL-6 and TNFα, key mediators in inflammatory processes, are central targets in immunotherapy due to their roles in tumor progression and immune modulation and their activity influencing nearly all biological processes, depending on the production levels and surrounding conditions [51]. Normalization of cytokine levels against untreated control cells revealed nuanced differences in their response to the treatments.

Our results indicated that single treatments with SAHA and 5-Aza-C strongly suppress both cytokines, indicating potential anti-inflammatory therapeutic benefits. Also, combinations involving SAHA + Cpt or 5-Aza-C + Ava led to significant increases in IL-6, highlighting a variable response in cytokine modulation, with TNFα generally showing greater suppression.

Global hypomethylation, a hallmark of tumor cells, promotes chromosomal instability and alters gene expression [52,53,54]. Notably, both effects appear to have a cis-regulatory nature, affecting genomic sites in close proximity to the hypomethylated regions [55,56]. If each LINE-1 locus exhibits unique methylation patterns, their roles in cellular biology and cancer development may vary based on their specific genomic locations. In our study, an increased methylation pattern was observed following treatment, with Ava presenting the most notable effects. Moreover, an inverse correlation was noticed between the downregulation of *EZH2* gene expression and the increase in the global LINE-1 methylation level. Alongside this modification, it is important to highlight the increase in *DNMT1* gene expression levels following the treatment with Avastin.

*EZH2* has been identified as a key player in cancer development and progression with its first reported involvement in oncogenesis linked to prostate cancer in 2002, by Varambally and colleagues [57]. Their research revealed that *EZH2* upregulation correlates with advanced disease stages and poor outcomes in prostate cancer. Subsequent studies demonstrated that *EZH2* is overexpressed in several solid malignancies, including lung, hepatocellular, colorectal, breast, and pancreatic cancers [58,59]. Additionally, *EZH2* has been shown to directly influence DNA methylation, contributing to the silencing of tumor suppressor genes in cancer [60].

We hypothesize that Avastin may increase methylation at the global level by activating the PRC 2 (Polycomb Repressive Complex 2) and *DNMT1*, but this molecular mechanism needs further investigations.

*DNMT3A* and *DNMT3B* gene expression levels were downregulated by the majority of treatment combinations, with Cpt + SAHA being notable relative to untreated cells and SAHA alone being notable relative to Cpt. Interestingly, the treatment with Ava determined a significant upregulation of DNMTs when compared with untreated cells.

Qiu et al. demonstrated that increased *DNMT3A* and *DNMT3B* gene expression correlates with cisplatin resistance in murine neuroblastoma cells, while platinum resistance remains a major obstacle in ovarian cancer treatment with Carboplatin or cisplatin, emphasizing the need for further exploration of the underlying molecular mechanisms [61,62].

In a study of 77 recurrent glioblastoma patients, Urup et al. presented that low methylation of the CEBP binding region in the *AGT* promoter is significantly associated with poor response to Bevacizumab (Avastin/Ava) combination treatment, while improved response to this therapy is correlated with enhanced survival [63].

To the best of our knowledge, this is the first study to mention the role of CPt and Ava in an in vitro model regarding the modulation of studied epigenetic factors.

Regarding the lncRNA expression level, the present study identified a significant reduction in *H19* gene expression levels following treatment with CPt + SAHA, Qct + SAHA, or Qct + 5-Aza C.

*H19* is known for its abnormal expression in various tumors, where it plays a role in promoting oncogenic processes including growth, migration, invasion, metastasis, epithelial–mesenchymal transition, autophagy, and cell cycle progression [64,65,66]. Furthermore, *H19* is associated with key clinical factors such as tumor size, clinical stage, lymph node metastasis, distant metastasis, and overall survival (OS), with studies suggesting its potential as biomarker in the progression of tumors [45,67]. A study by Liu et al. demonstrates that *H19* contributes to glioma progression by promoting cell proliferation, regulating the cell cycle, enhancing cell migration, and increasing the sphere-forming ability of glioma cell lines. This suggests that *H19* could serve as a potential biomarker for the diagnosis and treatment for glioma diagnosis and treatment [68].

*EMX2OS*, a tumor suppressor in several cancers, is strongly downregulated in follicular and papillary thyroid cancer, but also has not been intensely studied in the glioblastoma context [69,70]. In the present study, *EMX2OS* showed variable expression modulation with decreased expression following CPt and SAHA treatment and increased expression with CPt and 5-Aza C.

Another lncRNA investigated, *MALAT1*, has been found to undergo copy number alterations, translocations, or mutations in various types of cancer [71]. It has been identified as an RNA with elevated expression in more than 20 different solid and lymphoid tumors, where its overexpression (between 1.5- and 10-fold) has been associated with tumor progression and metastasis [72,73,74,75,76,77,78].

Conflicting evidence exists regarding its role in gliomas, with studies using established cell lines (U87, U251, SHG139) suggesting an anti-tumorigenic function, while research on patient-derived primary GBM cultures indicates a pro-tumorigenic role [79,80,81,82]. It is well known that established cell lines often lose the inherent molecular and pathophysiological characteristics of the original tumor, which may account for these discrepancies and highlight the limitations of cell culture conditions. However, in our study, *MALAT1* gene expression varied by treatment, increasing in three combinations and significantly decreasing in four, notably with Ava and SAHA, underscoring the need for further research using primary GBM cultures to clarify its functional role.

*ZFAS1*, *HAR1B*, and *HOTTIP* presented downregulated gene expression across all treatment combinations, with 5-Aza C having the most significant effect on *ZFAS1* and the combination of CPt + SAHA affecting *HAR1B* and *HOTTIP* most prominently. While the roles of these lncRNAs in glioblastoma cells are not well established, *ZFAS1* has been shown to promote glioma progression by enhancing proliferation, migration, and invasion and increase resistance to temozolomide in vitro through the miR-150-5p/PLP2 axis, as well as by regulating EMT and Notch signaling pathways [83,84].

Evidence suggests that *HAR1B’s* knockdown promotes the migration and invasion of gliomas, while others reported an increased resistance to treatment with pazopanib in sarcoma [85,86]. The studies reviewed by Waters et al. indicated that *HAR1A* and *HAR1B* may function as oncogenes or tumor suppressors depending on the cancer type, with their expression levels serving as potential prognostic biomarkers for tumor progression, particularly for gliomas and oral carcinomas [87].

*HOTTIP* plays critical roles in cancer cell growth, survival, migration, and invasion. Studies shown that its overexpression induces apoptosis and inhibits cell growth in U118 and U87 glioma cells by downregulating BRE gene expression, which influences the levels of p53, CDK2, and Cyclin A proteins, while elevated *HOTTIP* levels in temozolomide-resistant glioma cell lines A172, LN229, and SF268 enhance proliferation, migration, clonogenicity, and markers of angiogenesis and metastasis, highlighting its potential as therapeutic target [88,89].

*NRON*, known for its role in cancer-related immune responses, including T-cell activation, showed downregulated expression in our study, with the most significant decrease observed in the CPt + SAHA treatment combination, despite the limited literature on its involvement in glioblastoma [90].

*TUG1*, *MEG3*, *GAS5*, and *HOTAIR* did not exhibit significant changes in gene expression in this study. though prior research highlights their roles in glioma tumorigenesis and potential as biomarkers, particularly in the context of *HOTTIP*.

Studies have shown that *TUG1* and *MEG3* expression levels are reduced in human glioma tissues, with *TUG1* promoting cell apoptosis and suppressing proliferation, migration, and invasion, while *GAS5* was identified as a suppressor that enhances glioma therapy efficacy by directly targeting miR-222. Both in vivo and in vitro results demonstrated the therapeutic efficacy of GAS5-based gene therapy against glioma [91,92,93].

*HOTAIR* has been identified as a potential biomarker for glioma subtypes and tumor grades, with its silencing in glioblastoma cell lines (LN229 and U87) shown to disrupt cell cycle regulation by downregulating E2F1, cyclins, CDKs while upregulating inhibitors p16 and p21 [94].

Most of the lncRNAs studied exhibited decreased expression under combined treatments with CPt and SAHA, or Ava and SAHA, with SAHA playing a key role in moderating the reduction compared to treatments without it.

The correlation analysis highlights key relationships in gene expression profiles across different treatment conditions, shedding light on potential mechanisms of action and interactions between drugs. SAHA treatment consistently displayed strong positive correlations with several combinations, such as Ava + 5-Aza C and Ava + SAHA, suggesting a complementary effect on gene regulation when used with these therapies. Interestingly, SAHA also exhibited negative correlations with CPt and its combinations, indicating a potential antagonistic interaction. Ava treatment demonstrated a similar gene expression profile to CPt + 5-Aza C, Ava + 5-Aza C, and Qct + SAHA, implying overlapping molecular pathways. While CPt + SAHA showed limited correlations, its positive relationship with Qct + 5-Aza C points to a context-dependent interaction. The absence of significant correlations for 5-Aza C alone suggests its effects are more pronounced in combination therapies. These findings underline the complexity of drug interactions and the importance of exploring combination treatments to optimize therapeutic strategies.

The increase in global DNA methylation induced by CPt and Ava treatments suggests a role in silencing oncogenes and improving genome stability, though resistance to CPt remains a critical challenge. Beyond genetic alterations, epigenetic modifications of tumor suppressor genes involved in apoptosis, DNA repair, and cell cycle regulation may be key drivers of drug resistance.

Additionally, combinations like SAHA + CPt and Qct, or 5-Aza C + Ava significantly increased IL-6 levels, alongside varying effects on TNF-α. These findings suggest that histone deacetylase inhibitors, such as SAHA, may act as potent mediators in enhancing the efficacy of CPt and Ava treatments.

## 5. Conclusions

Our findings suggest a relationship between CPt and Ava treatments and DNA methylation mechanisms involving *EZH2*, *DNMT3A*, and *DNMT3B*. The direct effects of Ava on the expression levels of these specific DNA methyltransferases remain an area requiring further investigation.

The presented study offers significant insights into platinum-based therapies, establishing a foundation for the effective use of epigenetic drugs in combination with platinum agents. Looking ahead, combining platinum therapies with epigenetic drugs could pave the way for personalized treatments that overcome resistance and enhance therapeutic responses.

Notably, combined administration, particularly with HDAC inhibitors, shows promise in reactivating silenced tumor suppressor genes. Furthermore, the observed changes in methylation profiles in the in vitro model have spurred the testing of new combination treatment regimens in animal models. Given that certain HDAC inhibitors are already approved for human use, these findings hold potential for translating into clinical applications.

These findings contribute to a growing body of evidence suggesting that combinatorial strategies involving epigenetic modulators and cytostatics could overcome therapeutic resistance and improve outcomes in glioblastoma treatment. The differential effects of single and combined treatments on cytokine regulation in glioma cells suggests potential applications for tailoring therapies based on cytokine modulation profiles. Moreover, exploring the mechanisms driving these cytokine-specific responses could optimize therapeutic strategies in glioblastoma treatment.

## Figures and Tables

**Table 1:** Effects of drug treatments on the induction of early vs. late apoptosis.

	Early Apoptosis (%)	Late Apoptosis (%)	Total Apoptosis (%)
NT	4.2	1.6	5.8
SAHA (5 μM)	6.4	6.3	12.7
5-Aza C (5 μM)	8.4	5.2	13.6
CPt (50 μM)	5.6	7.7	13.3
Pxl (1 μM)	11.2	8.6	19.8
Ava (20 μg/mL)	11.6	9.5	21.1
Qct (50 μM)	16	10.8	26.8
SAHA + CPt	6.9	16	22.9
SAHA + Pxl	11.1	11.2	22.3
SAHA + Ava	5.6	13	18.6
SAHA + Qct	8.7	16.5	25.2
5-Aza C + CPt	10.1	11.4	21.5
5-Aza C + Pxl	5.7	9.7	15.4
5-Aza C + Ava	4.2	12	16.2
5-Aza C + Qct	5.7	13.6	19.3

## Data Availability

The original contributions presented in the study are included in the article/Supplementary Materials; further inquiries can be directed to the corresponding author.

## Supplementary Materials

The following supporting information can be downloaded at https://www.mdpi.com/article/10.3390/genes16020138/s1: Figure S1. lncRNA profiler array in untreated U87-MG cells; Figure S2. Induction of apoptosis in U87-MG glioma cells by single vs. combined treatments; Table S1. Primer sequences for the investigated target genes; Table S2. Evaluation of DNA progression through U87-MG cell cycle phases after single or combined drug treatments; Table S3. Unpaired *t*-test *p*-values regarding mRNA expression levels of selected genes, modulated in different treatments. Bold—significant *p*-value (*p* < 0.05); Table S4. LINE-1 global methylation assay in treated and untreated U87-MG cells.

## References

[1] Stupp R., Hegi M.E., Mason W.P., Van Den Bent M.J., Taphoorn M.J., Janzer R.C., Ludwin S.K., Allgeier A., Fisher B., Belanger K. (2009). Effects of radiotherapy with concomitant and adjuvant temozolomide versus radiotherapy alone on survival in glioblastoma in a randomised phase III study: 5-year analysis of the EORTC-NCIC trial. Lancet Oncol..

[2] Bray F., Laversanne M., Sung H., Ferlay J., Siegel R.L., Soerjomataram I., Jemal A. (2024). Global cancer statistics 2022: GLOBOCAN estimates of incidence and mortality worldwide for 36 cancers in 185 countries. CA Cancer J. Clin..

[3] Cho H., Mariotto A.B., Schwartz L.M., Luo J., Woloshin S. (2014). When do changes in cancer survival mean progress? The insight from population incidence and mortality. J. Natl. Cancer Inst. Monogr..

[4] Cancer Trends Progress Report National Cancer Institute; NIH, DHHS, Bethesda, MD. March 2024. https://progressreport.cancer.gov.

[5] Krex D., Klink B., Hartmann C., von Deimling A., Pietsch T., Simon M., Sabel M., Steinbach J.P., Heese O., Reifenberger G. (2007). Long-term survival with glioblastoma multiforme. Brain.

[6] Brodbelt A., Greenberg D., Winters T., Williams M., Vernon S., Collins V.P., (UK) National Cancer Information Network Brain Tumour Group (2015). Glioblastoma in England: 2007–2011. Eur. J. Cancer.

[7] Grochans S., Cybulska A.M., Simińska D., Korbecki J., Kojder K., Chlubek D., Baranowska-Bosiacka I. (2022). Epidemiology of Glioblastoma Multiforme-Literature Review. Cancers.

[8] Ostrom Q.T., Bauchet L., Davis F.G., Deltour I., Fisher J.L., Langer C.E., Pekmezci M., Schwartzbaum J.A., Turner M.C., Walsh K.M. (2014). The epidemiology of glioma in adults: A “state of the science” review. Neuro Oncol..

[9] Zreik J., Moinuddin F.M., Yolcu Y.U., Alvi M.A., Chaichana K.L., Quinones-Hinojosa A., Bydon M. (2020). Improved 3-year survival rates for glioblastoma multiforme are associated with trends in treatment: Analysis of the national cancer database from 2004 to 2013. J. Neurooncol..

[10] Ostrom Q.T., Patil N., Cioffi G., Waite K., Kruchko C., Barnholtz-Sloan J.S. (2020). CBTRUS Statistical Report: Primary Brain and Other Central Nervous System Tumors Diagnosed in the United States in 2013–2017. Neuro Oncol..

[11] Wu W., Klockow J.L., Zhang M., Lafortune F., Chang E., Jin L., Wu Y., Daldrup-Link H.E. (2021). Glioblastoma multiforme (GBM): An overview of current therapies and mechanisms of resistance. Pharmacol. Res..

[12] Gallego O. (2015). Nonsurgical treatment of recurrent glioblastoma. Curr. Oncol..

[13] Huang Z., Cheng L., Guryanova O.A., Wu Q., Bao S. (2010). Cancer stem cells in glioblastoma-molecular signaling and therapeutic targeting. Protein Cell.

[14] Tamimi A.F., Juweid M., De Vleeschouwer S. (2017). Epidemiology and Outcome of Glioblastoma. Glioblastoma [Internet].

[15] Yan H., Parsons D.W., Jin G., McLendon R., Rasheed B.A., Yuan W., Kos I., Batinic-Haberle I., Jones S., Riggins G.J. (2009). IDH1 and IDH2 mutations in gliomas. N. Engl. J. Med..

[16] Louis D.N., Perry A., Wesseling P., Brat D.J., Cree I.A., Figarella-Branger D., Hawkins C., Ng H.K., Pfister S.M., Reifenberger G. (2021). The 2021 WHO Classification of Tumors of the Central Nervous System: A summary. Neuro Oncol..

[17] Brennan C.W., Verhaak R.G., McKenna A., Campos B., Noushmehr H., Salama S.R., Zheng S., Chakravarty D., Sanborn J.Z., Berman S.H. (2013). The somatic genomic landscape of glioblastoma. Cell.

[18] Capper D., Jones D.T.W., Sill M., Hovestadt V., Schrimpf D., Sturm D., Koelsche C., Sahm F., Chavez L., Reuss D.E. (2018). DNA methylation-based classification of central nervous system tumours. Nature.

[19] Ceccarelli M., Barthel F.P., Malta T.M., Sabedot T.S., Salama S.R., Murray B.A., Morozova O., Newton Y., Radenbaugh A., Pagnotta S.M. (2016). Molecular Profiling Reveals Biologically Discrete Subsets and Pathways of Progression in Diffuse Glioma. Cell.

[20] Sturm D., Witt H., Hovestadt V., Khuong-Quang D.A., Jones D.T., Konermann C., Pfaff E., Tönjes M., Sill M., Bender S. (2012). Hotspot mutations in H3F3A and IDH1 define distinct epigenetic and biological subgroups of glioblastoma. Cancer Cell.

[21] Albulescu A., Plesa A., Fudulu A., Iancu I.V., Anton G., Botezatu A. (2021). Epigenetic approaches for cervical neoplasia screening (Review). Exp. Ther. Med..

[22] Chen J., Ao L., Yang J. (2019). Long non-coding RNAs in diseases related to inflammation and immunity. Ann. Transl. Med..

[23] Richard Boland C. (2017). Non-coding RNA: It’s Not Junk. Dig. Dis. Sci..

[24] Peng Z., Liu C., Wu M. (2018). New insights into long noncoding RNAs and their roles in glioma. Mol. Cancer.

[25] Choudhari R., Sedano M.J., Harrison A.L., Subramani R., Lin K.Y., Ramos E.I., Lakshmanaswamy R., Gadad S.S. (2020). Long noncoding RNAs in cancer: From discovery to therapeutic targets. Adv. Clin. Chem..

[26] Bjørklund S.S., Aure M.R., Häkkinen J., Vallon-Christersson J., Kumar S., Evensen K.B., Fleischer T., Tost J., Osbreac, Bathen T.F. (2022). Subtype and cell type specific expression of lncRNAs provide insight into breast cancer. Commun. Biol..

[27] Sousa G.F., Wlodarczyk S.R., Monteiro G. (2014). Carboplatin: Molecular mechanisms of action associated with chemoresistance. Braz. J. Pharm..

[28] Jatoi A., Martenson J.A., Foster N.R., McLeod H.L., Lair B.S., Nichols F., Tschetter L.K., Moore DFJr Fitch T.R., Alberts S.R., North Central Cancer Treatment Group (N0044) (2007). Paclitaxel, carboplatin, 5-fluorouracil, and radiation for locally advanced esophageal cancer: Phase II results of preliminary pharmacologic and molecular efforts to mitigate toxicity and predict outcomes: North Central Cancer Treatment Group (N0044). Am. J. Clin. Oncol..

[29] Garcia J., Hurwitz H.I., Sandler A.B., Miles D., Coleman R.L., Deurloo R., Chinot O.L. (2020). Bevacizumab (Avastin^®^) in cancer treatment: A review of 15 years of clinical experience and future outlook. Cancer Treat. Rev..

[30] Vafadar A., Shabaninejad Z., Movahedpour A., Fallahi F., Taghavipour M., Ghasemi Y., Akbari M., Shafiee A., Hajighadimi S., Moradizarmehri S. (2020). Quercetin and cancer: New insights into its therapeutic effects on ovarian cancer cells. Cell Biosci..

[31] Albulescu A., Fudulu A., Botezatu A., Iancu I.V., Plesa A., Brasoveanu L.I. (2021). Long noncoding RNA profile in neuroblastoma and glioblastoma. FEBS Open Bio.

[32] Albulescu A., Petrescu G.E.D., Botezatu A., Fudulu A., Iancu I.V., Plesa A., Radu R., Brehar F.M., Gorgan R.M., Brasoveanu L.I. lncRNA Profile in Glioblastoma Samples and Cell Lines. “2nd OncoHUB Conference”. Proceedings of the 2nd Edition of the OncoHub Conference—Connecting Scientists and Physicians for Next Generation.

[33] Mihaila M., Bostan M., Hotnog D., Ferdes M., Brasoveanu L.I. (2013). Real-time analysis of quercetin, resveratrol and/or doxorubicin effects in MCF-7 cells. Rom. Biotechnol. Lett..

[34] Bostan M., Petrica-Matei G.G., Ion G., Radu N., Mihaila M., Hainarosie R., Brasoveanu L.I., Roman V., Constantin C., Neagu M.T. (2019). Cisplatin effect on head and neck squamous cell carcinoma cells is modulated by ERK1/2 protein kinases. Exp. Ther. Med..

[35] Munteanu A.-C., Badea M., Olar R., Silvestro L., Mihaila M., Brasoveanu L.I., Musat M.G., Andries A., Uivarosi V. (2018). Cytotoxicity studies, DNA interaction and protein binding of new Al (III), Ga (III) and In (III) complexes with 5-hydroxyflavone. Appl. Organometal Chem..

[36] Munteanu A., Musat M.G., Mihaila M., Badea M., Olar R., Nitulescu G.M., Rădulescu F.Ș., Brasoveanu L.I., Uivarosi V. (2021). New Heteroleptic Lanthanide Complexes as Multimodal Drugs: Cytotoxicity Studies, Apoptosis, Cell Cycle Analysis, DNA Interactions, and Protein Binding. Appl. Organomet. Chem..

[37] Iancu I.V., Botezatu A., Plesa A., Huica I., Fudulu A., Albulescu A., Bostan M., Mihaila M., Grancea C., Manda D.A. (2020). Alterations of regulatory factors and DNA methylation pattern in thyroid cancer. Cancer Biomark..

[38] Ivan B.-C., Barbuceanu S.-F., Hotnog C.M., Anghel A.I., Ancuceanu R.V., Mihaila M.A., Brasoveanu L.I., Shova S., Draghici C., Olaru O.T. (2022). New Pyrrole Derivatives as Promising Biological Agents: Design, Synthesis, Characterization, In Silico, and Cytotoxicity Evaluation. Int. J. Mol. Sci..

[39] Maciuca A.-M., Munteanu A.-C., Mihaila M., Badea M., Olar R., Nitulescu G.M., Munteanu C.V.A., Bostan M., Uivarosi V. (2020). Rare-Earth Metal Complexes of the Antibacterial Drug Oxolinic Acid: Synthesis, Characterization, DNA/Protein Binding and Cytotoxicity Studies. Molecules.

[40] Fischer V., Kalbitz M., Müller-Graf F., Gebhard F., Ignatius A., Liedert A., Haffner-Luntzer M. (2018). Influence of menopause on inflammatory cytokines during murine and human bone fracture healing. Int. J. Mol. Sci..

[41] Hotnog D., Mihaila M., Botezatu A., Matei G.G., Hotnog C., Anton G., Bostan M., Brasoveanu L.I. (2013). Genistein potentiates the apoptotic effect of 5-fluorouracyl in colon cancer cell lines. Rom. Biotechnol. Lett..

[42] Feng Y., Kang Y., He Y., Liu J., Liang B., Yang P., Yu Z. (2014). MicroRNA-99a acts as a tumor suppressor and is down-regulated in bladder cancer. BMC Urol..

[43] Mohammadi K., Baghini S.S., Saremi M.A. (2020). Increased expression of the lncH19 gene in the plasma of people with breast cancer. Pers. Med..

[44] Hotoboc I.E., Fudulu A., Grigore R., Bertesteanu S., Huica I., Iancu I.V., Botezatu A., Bleotu C., Anton G. (2021). The association between lncRNA H19 and EZH2 expression in patients with EBV-positive laryngeal carcinoma. Acta Otorhinolaryngol. Ital..

[45] Panoilia E., Schindler E., Samantas E., Aravantinos G., Kalofonos H.P., Christodoulou C., Patrinos G.P., Friberg L.E., Sivolapenko G. (2015). A pharmacokinetic binding model for bevacizumab and VEGF165 in colorectal cancer patients. Cancer Chemother. Pharmacol..

[46] Albulescu A., Petrescu G.E.D., Fudulu A., Botezatu A., Iancu I.V., Pleșa A., Dragomir M.P., Pașov D., Cocoșilă L., Brehar F.M. Expression Analysis of Key Epigenetic Regulators in Glioblastoma. Proceedings of the International Pathology Conference of the Victor Babes Institute.

[47] Townson J.L., Naumov G.N., Chambers A.F. (2003). The role of apoptosis in tumor progression and metastasis. Curr. Mol. Med..

[48] Su Z., Yang Z., Xu Y., Chen Y., Yu Q. (2015). Apoptosis, autophagy, necroptosis, and cancer metastasis. Mol. Cancer.

[49] Pfeffer C.M., Singh A.T.K. (2018). Apoptosis: A target for anticancer therapy. Int. J. Mol. Sci..

[50] Kim H.I., Lee S.J., Choi Y.J., Kim M.J., Kim T.Y., Ko S.G. (2021). Quercetin Induces Apoptosis in Glioblastoma Cells by Suppressing Axl/IL-6/STAT3 Signaling Pathway. Am. J. Chin. Med..

[51] Soler M.F., Abaurrea A., Azcoaga P., Araujo A.M., Caffarel M.M. (2023). New perspectives in cancer immunotherapy: Targeting IL-6 cytokine family. J. Immunother. Cancer.

[52] Lengauer C., Kinzler K.W., Vogelstein B. (1997). DNA methylation and genetic instability in colorectal cancer cells. Proc. Natl. Acad. Sci. USA.

[53] Eden A., Gaudet F., Waghmare A., Jaenisch R. (2003). Chromosomal instability and tumors promoted by DNA hypomethylation. Science.

[54] Hellman A., Chess A. (2007). Gene body-specific methylation on the active X chromosome. Science.

[55] Qu G.Z., Grundy P.E., Narayan A., Ehrlich M. (1999). Frequent hypomethylation in Wilms tumors of pericentromeric DNA in chromosomes 1 and 16. Cancer Genet. Cytogenet..

[56] Pornthanakasem W., Kongruttanachok N., Phuangphairoj C., Suyarnsestakorn C., Sanghangthum T., Oonsiri S., Ponyeam W., Thanasupawat T., Matangkasombut O., Mutirangura A. (2008). LINE-1 methylation status of endogenous DNA double-strand breaks. Nucleic Acids Res..

[57] Varambally S., Dhanasekaran S.M., Zhou M., Barrette T.R., Kumar-Sinha C., Sanda M.G., Ghosh D., Pienta K.J., Sewalt R.G., Otte A.P. (2002). The polycomb group protein EZH2 is involved in progression of prostate cancer. Nature.

[58] Pasini D., Emerging D.C.L. (2016). Roles for Polycomb proteins in cancer. Curr. Opin. Genet. Dev..

[59] Yamagishi M., Uchimaru K. (2017). Targeting EZH2 in cancer therapy. Curr. Opin. Oncol..

[60] Kodach L.L., Jacobs R.J., Heijmans J., van Noesel C.J., Langers A.M., Verspaget H.W., Hommes D.W., Offerhaus G.J., van den Brink G.R., Hardwick J.C. (2010). The role of EZH2 and DNA methylation in the silencing of the tumour suppressor RUNX3 in colorectal cancer. Carcinogenesis.

[61] Qiu Y.Y., Mirkin B.L., Dwivedi R.S. (2005). Inhibition of DNA methyltransferase reverses cisplatin induced drug resistance in murine neuroblastoma cells. Cancer Detect. Prev..

[62] Schwarzenbach H., Gahan P.B. (2019). Resistance to cis- and carboplatin initiated by epigenetic changes in ovarian cancer patients. Cancer Drug Resist..

[63] Urup T., Gillberg L., Kaastrup K., Lü M.J.S., Michaelsen S.R., Andrée Larsen V., Christensen I.J., Broholm H., Lassen U., Grønbaek K. (2020). Angiotensinogen promoter methylation predicts bevacizumab treatment response of patients with recurrent glioblastoma. Mol. Oncol..

[64] Wang G., Lin X., Han H., Zhang H., Li X., Feng M., Jiang C. (2022). lncRNA H19 promotes glioblastoma multiforme development by activating autophagy by sponging miR-491-5p. Bioengineered.

[65] Liang W.Q., Zeng D., Chen C.F., Sun S.M., Lu X.F., Peng C.Y., Lin H.Y. (2019). Long noncoding RNA H19 is a critical oncogenic driver and contributes to epithelial-mesenchymal transition in papillary thyroid carcinoma. Cancer Manag. Res..

[66] Wu B., Zhang Y., Yu Y., Zhong C., Lang Q., Liang Z., Lv C., Xu F., Tian Y. (2021). Long Noncoding RNA H19: A Novel Therapeutic Target Emerging in Oncology Via Regulating Oncogenic Signaling Pathways. Front. Cell Dev. Biol..

[67] Li H., Yu B., Li J., Su L., Yan M., Zhu Z., Liu B. (2014). Overexpression of lncRNA H19 enhances carcinogenesis and metastasis of gastric cancer. Oncotarget.

[68] Liu P., Huang X., Wu H., Yin G., Shen L. (2021). LncRNA-H19 gene plays a significant role in regulating glioma cell function. Mol. Genet. Genomic Med..

[69] Zhang H.M., Cui M.Y., Chen Z.H. (2023). EMX2OS targeting IGF2BP1 represses Wilms’ tumour stemness, epithelial-mesenchymal transition and metastasis. J. Genet..

[70] Gu Y., Feng C., Liu T., Zhang B., Yang L. (2018). The downregulation of lncRNA EMX2OS might independently predict shorter recurrence-free survival of classical papillary thyroid cancer. PLoS ONE.

[71] Davis I.J., Hsi B.L., Arroyo J.D., Vargas S.O., Yeh Y.A., Motyckova G., Valencia P., Perez-Atayde A.R., Argani P., Ladanyi M. (2003). Cloning of an α-TFEB fusion in renal tumors harboring the t(6;11)(p21;q13) chromosome translocation. Proc. Natl. Acad. Sci. USA.

[72] Arun G., Diermeier S., Akerman M., Chang K.-C., Wilkinson J.E., Hearn S., Kim Y., MacLeod A.R., Krainer A.R., Norton L. (2016). Differentiation of mammary tumors and reduction in metastasis upon Malat1 lncRNA loss. Genes. Dev..

[73] Amodio N., Stamato M.A., Juli G., Morelli E., Fulciniti M., Manzoni M., Taiana E., Agnelli L., Cantafio M.E.G., Romeo E. (2018). Drugging the lncRNA MALAT1 via LNA gapmeR ASO inhibits gene expression of proteasome subunits and triggers anti-multiple myeloma activity. Leukemia.

[74] Ying L., Chen Q., Wang Y., Zhou Z., Huang Y., Qiu F. (2012). Upregulated MALAT-1 contributes to bladder cancer cell migration by inducing epithelial-to-mesenchymal transition. Mol. Biosyst..

[75] Guo F., Liu Y., Li Y., Li G. (2010). Inhibition of ADP-ribosylation factor-like 6 interacting protein 1 suppresses proliferation and reduces tumor cell invasion in CaSki human cervical cancer cells. Mol. Biol. Rep..

[76] Shen L., Chen L., Wang Y., Jiang X., Xia H., Zhuang Z. (2015). Long noncoding RNA MALAT1 promotes brain metastasis by inducing epithelial-mesenchymal transition in lung cancer. J. Neurooncol.

[77] Wang X., Li M., Wang Z., Han S., Tang X., Ge Y., Zhou L., Zhou C., Yuan Q., Yang M. (2015). Silencing of long noncoding RNA MALAT1 by miR-101 and miR-217 inhibits proliferation, migration, and invasion of esophageal squamous cell carcinoma cells. J. Biol. Chem..

[78] Gutschner T., Hammerle M., Eissmann M., Hsu J., Kim Y., Hung G., Revenko A., Arun G., Stentrup M., Gross M. (2013). The Noncoding RNA MALAT1 Is a Critical Regulator of the Metastasis Phenotype of Lung Cancer Cells. Cancer Res..

[79] Han Y., Wu Z., Wu T., Huang Y., Cheng Z., Li X., Sun T., Xie X., Zhou Y., Du Z. (2016). Tumor-suppressive function of long noncoding RNA MALAT1 in glioma cells by downregulation of MMP2 and inactivation of ERK/MAPK signaling. Cell Death Dis..

[80] Han Y., Zhou L., Wu T., Huang Y., Cheng Z., Li X., Sun T., Zhou Y., Du Z. (2016). Downregulation of lncRNA-MALAT1 Affects Proliferation and the Expression of Stemness Markers in Glioma Stem Cell Line SHG139S. Cell Mol. Neurobiol..

[81] Cao S., Wang Y., Li J., Lv M., Niu H., Tian Y. (2016). Tumor-suppressive function of long noncoding RNA MALAT1 in glioma cells by suppressing miR-155 expression and activating FBXW7 function. Am. J. Cancer Res..

[82] Voce D.J., Bernal G.M., Wu L., Crawley C.D., Zhang W., Mansour N.M., Cahill K.E., Szymura S.J., Uppal A., Raleigh D.R. (2019). Temozolomide Treatment Induces lncRNA MALAT1 in an NF-κB and p53 Codependent Manner in Glioblastoma. Cancer Res..

[83] Li X., Luo Y., Liu L., Cui S., Chen W., Zeng A., Shi Y., Luo L. (2020). The long noncoding RNA ZFAS1 promotes the progression of glioma by regulating the miR-150-5p/PLP2 axis. J. Cell Physiol..

[84] Gao K., Ji Z., She K., Yang Q., Shao L. (2017). Long non-coding RNA ZFAS1 is an unfavourable prognostic factor and promotes glioma cell progression by activation of the Notch signaling pathway. Biomed. Pharmacother..

[85] Huang K., Yue X., Zheng Y., Zhang Z., Cheng M., Li L., Chen Z., Yang Z., Bian E., Zhao B. (2021). Development and Validation of an Mesenchymal-Related Long Non-Coding RNA Prognostic Model in Glioma. Front. Oncol..

[86] Yamada H., Takahashi M., Watanuki M., Watanabe M., Hiraide S., Saijo K., Komine K., Ishioka C. (2021). lncRNA HAR1B has potential to be a predictive marker for pazopanib therapy in patients with sarcoma. Oncol. Lett..

[87] Waters E., Pucci P., Hirst M., Chapman S., Wang Y., Crea F., Heath C.J. (2021). HAR1: An insight into lncRNA genetic evolution. Epigenomics.

[88] Xu L.M., Chen L., Li F., Zhang R., Li Z.Y., Chen F.F., Jiang X.D. (2016). Over-expression of the long non-coding RNA HOTTIP inhibits glioma cell growth by BRE. J. Exp. Clin. Cancer Res..

[89] Li Z., Li M., Xia P., Lu Z. (2022). HOTTIP Mediated Therapy Resistance in Glioma Cells Involves Regulation of EMT-Related miR-10b. Front Oncol.

[90] Chae Y., Roh J., Kim W. (2021). The Roles Played by Long Non-Coding RNAs in Glioma Resistance. Int. J. Mol. Sci..

[91] Li J., Zhang M., An G., Ma Q. (2016). LncRNA TUG1 acts as a tumor suppressor in human glioma by promoting cell apoptosis. Exp. Biol. Med..

[92] Zhang S., Guo W. (2019). Long noncoding RNA MEG3 suppresses the growth of glioma cells by regulating the miR-96-5p/MTSS1 signaling pathway. Mol. Med. Rep..

[93] Zhao X., Wang P., Liu J., Zheng J., Liu Y., Chen J., Xue Y. (2015). Gas5 exerts tumor-suppressive functions in human glioma cells by targeting miR-222. Mol. Ther..

[94] Zhang J.-X., Han L., Bao Z.-S., Wang Y.-Y., Chen L.-Y., Yan W., Yu S.-Z., Pu P.-Y., Liu N., You Y.-P. (2013). HOTAIR, a cell cycle–associated long noncoding RNA and a strong predictor of survival, is preferentially expressed in classical and mesenchymal glioma. Neuro-Oncol..

