# Supplementary material for: Combined Effect of Conventional Chemotherapy with Epigenetic Modulators on Glioblastoma
Source: Genes (Basel). 2025 Jan 24;16(2):138. doi: 10.3390/genes16020138 (PMC11855767; doi:10.3390/genes16020138)
Supplement: Supplementary file 1 [file genes-16-00138-s001.zip › Supplementary Tables.pdf]

Table S1. Primer sequences for the investigated target genes

Target	Primer sequence	Reference
NRON	F: CATGGCGACGGCAAAATCAT	[This study]
	R: AACCCCCAAACCTTCCGATG	
EMX2OS	F: CTTGCACCAACCCTTTTCCG	[This study]
	R: CAGCCTAGACCACCTACCCT	
ZFAS1	F: ATTGTCCTGCCCGTTAGAGC	[This study]
	R: ACTTCCAACACCCGCATTCA	
HAR1B	F: ACGTCTCCTCCGTTTCATGC	[This study]
	R: TCAGACCTGGTTGCAGAGTG	
TUG1	F: CGACTGAGCAAGCACTACCA	[This study]
	R: CTCAGCAATCAGGAGGCACA	
HOTAIR	F: GGTAGAAAAAGCAACCACGAAGC	[This study]
	R: ACATAAACCTCTGTCTGTGAGTGCC	
GAS5	F: CTTCTGGGCTCAAGTGATCCT	[This study]
	R: TTGTGCCATGAGACTCCATCAG	
MEG3	F: ATCCTGCTGGCAACTCCAAG	[This study]
	R: GCGTGCCTTTGGTGATTCA	
HOTTIP	F: ACTTTCTGGCCGTTTCACCA	[This study]
	R: CAGGGGCCTAGAACCCTACT	
MALAT1	F: AAAGCAAGGTCTCCCCACAAG	[This study]
	R: GGTCTGTGCTAGATCAAAAGGCA	
U6	F: CTCGCTTCGGCAGCACATATACT	[40]
	R: ACGCTTCACGAATTTGCGTGTC	
H19	F: TGCTGCACTTTACAACCACTG	[41]
	R: ATGGTGTCTTTGATGTTGGGC	
EZH2	F: TGCAACACCCAACACTTATAAGCGG	[42]
	R: CCTTTGCTCCCTCCAAATGCTGGT	
DNMT1	F: GCGGTATACCCACCATGACA	[36]
	R: AGGCTTTGCCGGCTTCC	
DNMT3A	F: CTGACAGAGGCACCGTTCAC	[This study]
	R: TATCGTGGTCTTTGGAGGCG	
DNMT3B	F: CCAACAACACGCAACCAGTG	[This study]
	R: CGTCTTCGAGTCTTGTTCTCGTA	

Table S2. Evaluation of DNA progression through U87-MG cell cycle phases after single or combined drug treatments

Drug treatment	Cell cycle phases			Proliferation index
	G0/G1 (%)	S (%)	G2M (%)	S+G2M (%)
**NT**	70.55	25.64	3.81	29.45
**SAHA (5 µM)**	73.24	16.02	10.73	26.75
**5-Aza C (5 µM)**	73.94	16.67	9.39	26.06
**CPt (50 µM)**	20.87	32.85	46.28	79.13
**Pxl (1 µM)**	24.28	37.64	38.08	75,72
**Ava (20 µg/ml)**	72.07	17.62	10.31	27.93
**Qct (50 µM)**	60.57	30.98	8.45	39.43
**SAHA + CPt**	14.45	45.62	39.93	85.55
**SAHA + Pxl**	9.44	18.37	72.19	90.56
**SAHA + Ava**	72.94	16.87	10.19	27.06
**SAHA + Qct**	47.65	27.09	25.26	52.35
**5-Aza C + CPt**	20.17	42.72	37.11	79.83
**5-Aza C + Pxl**	18.27	0.49	81.24	81.73
**5-Aza C + Ava**	72.76	20.80	6.44	27.24
**5-Aza C + Qct**	61.46	28.65	9.89	38.54

Table S3. Unpaired t-test p-values regarding mRNA expression levels of selected genes, modulated in different treatments. Bold – significant p-value ($p<0.05$).

	NRON	EMX2OS	ZFAS1	HAR1B	TUG1	MALAT1	H19	MEG3	HOTTIP	EZH2	GAS5	HOTAIR	DNMT1	DNMT3A	DNMT3B
SAHA vs. NT	0.3044	0.3252	0.4765	0.1402	0.4198	**0.0342**	**0.0064**	0.6825	0.3641	0.1495	0.4557	0.5002	0.5197	**0.0005**	0.2827
5-Aza C vs. NT	0.4083	0.0679	**0.0001**	0.0793	0.6875	0.2846	0.0749	0.9329	0.1000	0.4493	0.4448	0.5680	**0.0473**	**0.0078**	0.7130
CPt vs. NT	0.3364	0.0698	**0.0955**	0.4946	0.1573	0.7232	**0.0028**	0.9838	0.4654	0.0137	0.9916	0.4348	**0.0178**	0.0717	**0.0001**
Ava vs. NT	0.1856	**0.0325**	**0.0220**	0.6295	0.2009	**0.0485**	**0.0008**	0.4860	0.4664	0.0105	0.7255	0.9841	**0.0001**	**0.0009**	**0.0001**
CPt + SAHA vs. NT	**0.0009**	**0.0001**	0.0869	**0.0004**	0.3688	0.0598	**0.0001**	0.1231	**0.0003**	0.0169	0.1056	0.8538	**0.0007**	**0.0001**	**0.0211**
CPt + SAHA vs. SAHA	0.1344	**0.0002**	0.1411	**0.0186**	0.1840	**0.0054**	**0.0029**	0.0567	**0.0005**	0.1027	0.3515	0.3779	**0.0015**	**0.0153**	**0.0031**
CPt + SAHA vs. CPt	**0.0016**	**0.0001**	0.7370	**0.0006**	**0.0664**	0.0860	**0.0314**	0.1177	**0.0061**	0.6150	0.0957	0.3344	**0.0001**	**0.0001**	**0.0014**
Ava + SAHA vs. NT	**0.0171**	**0.0066**	0.3433	**0.0016**	0.6688	**0.0244**	**0.0022**	0.7493	**0.0095**	**0.0299**	0.1422	0.1727	0.2851	**0.0016**	0.2649
Ava + SAHA vs. SAHA	0.3206	**0.0204**	0.5975	0.1211	0.7796	0.7098	0.2809	0.9094	**0.0301**	0.1499	0.5485	0.4231	0.6494	0.3490	0.9581
Ava + SAHA vs. Ava	**0.0089**	**0.0001**	0.0712	**0.0030**	0.5222	0.9687	0.8492	0.6813	0.1261	0.5081	0.3463	0.1544	**0.0002**	**0.0001**	**0.0003**
Qct + SAHA vs. NT	0.4484	0.7327	0.7707	0.2070	0.3416	**0.0316**	**0.0001**	0.8689	0.2554	**0.0268**	0.5527	0.7563	0.6299	0.8089	0.3131
Qct + SAHA vs. SAHA	0.8696	0.1740	0.7238	0.4113	0.9816	0.9927	**0.0010**	0.5492	0.6711	0.0779	0.9000	0.6906	0.2740	**0.0003**	0.9440
CPt + 5-Aza C vs. NT	0.6193	**0.0247**	0.0627	0.5650	0.1386	0.2693	**0.0003**	0.6062	0.3706	**0.0041**	0.5458	0.3144	0.6372	0.0679	**0.0014**
CPt + 5-Aza C vs. 5-Aza C	0.6517	**0.0003**	**0.0001**	0.1711	0.0895	0.0956	**0.0066**	0.6579	0.4880	**0.0090**	0.9109	0.5877	**0.0221**	0.0005	**0.0011**
CPt + 5-Aza C vs. CPt	0.3484	0.2697	0.3040	0.9918	0.9347	0.3829	0.7581	0.6196	0.9237	0.3888	0.5263	0.8210	**0.0085**	0.9729	**0.0001**
Ava + 5-Aza C vs. NT	0.0980	0.0965	0.7502	0.0790	0.4441	0.0626	**0.0034**	0.8903	**0.0265**	0.1137	0.3943	0.5692	0.2783	**0.0429**	0.1913
Ava + 5-Aza C vs. 5-Aza C	0.7190	0.8318	**0.0001**	0.7743	0.3218	0.2185	0.1102	0.9579	0.4392	0.2320	0.8539	0.9931	**0.0080**	0.2951	0.1162
Ava + 5-Aza C vs. Ava	**0.0483**	**0.0008**	**0.0296**	0.1191	0.7351	0.9834	0.2968	0.5537	0.1803	0.3515	0.6796	0.5374	**0.0002**	**0.0001**	**0.0004**
Qct + 5-Aza C vs. NT	0.0745	**0.0425**	0.1047	**0.0466**	0.9447	0.6495	**0.0005**	0.1272	0.0678	**0.0167**	0.6043	0.7190	**0.0017**	**0.0045**	**0.0663**

Table S4. LINE-1 Global methylation assay in treated and untreated U87-MG cells

	**Methylation (%)**
**NT**	**3.66**
**SAHA**	**51.45**
**5-Aza C**	**68.31**
**Cpt**	**47.54**
**Ava**	**85.87**
**Cpt + SAHA**	**70.97**
**Ava + SAHA**	**78.72**
**Qct + SAHA**	**72.33**
**Cpt + 5-Aza C**	**-**
**Ava + 5 Aza C**	**60.97**
**Qct + 5-Aza C**	**81.38**
