# Supplementary figures and images for: Combined Effect of Conventional Chemotherapy with Epigenetic Modulators on Glioblastoma
Source: Genes (Basel). 2025 Jan 24;16(2):138. doi: 10.3390/genes16020138 (PMC11855767; doi:10.3390/genes16020138)

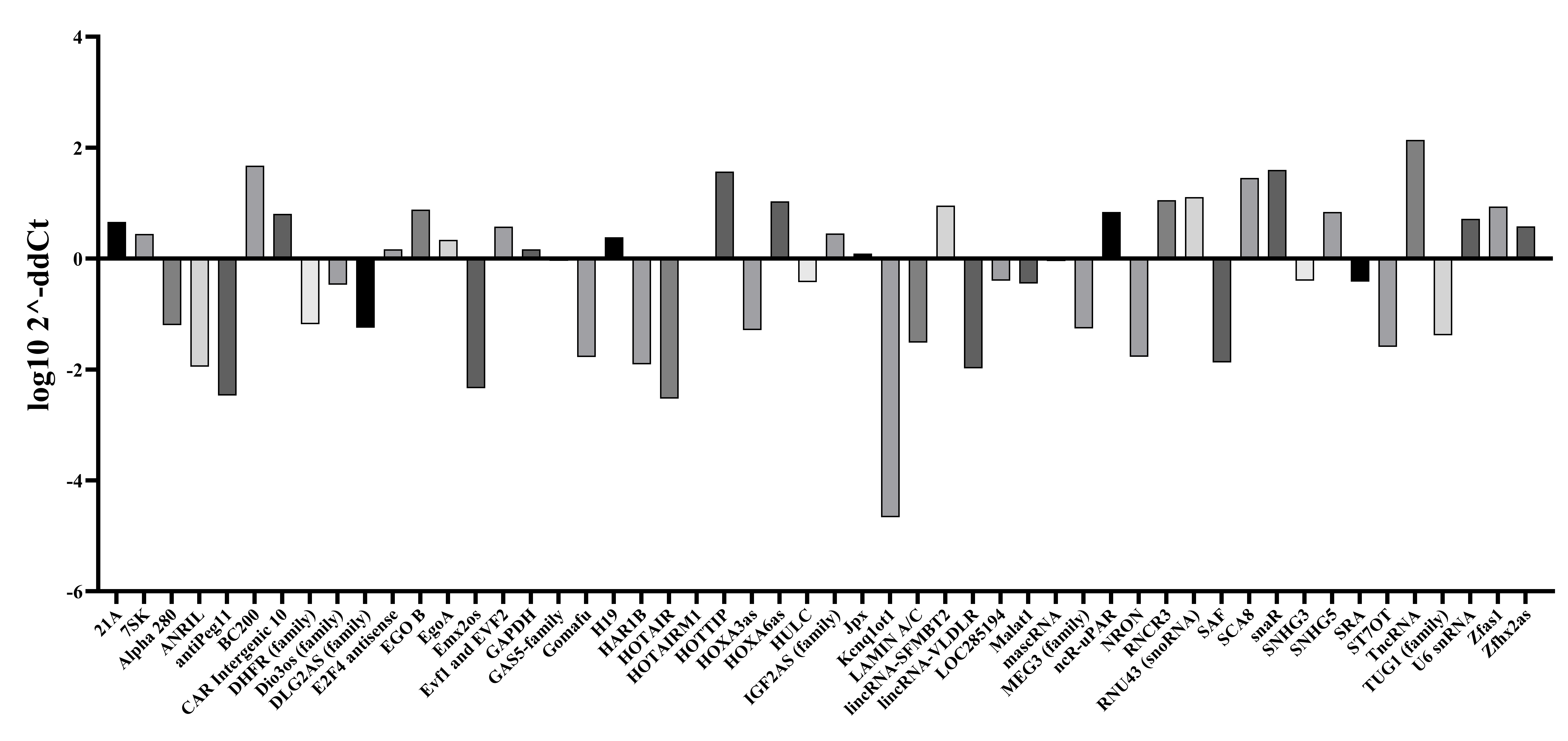

Supplement: Supplementary file 1 [file genes-16-00138-s001.zip › Fig S1.tif]

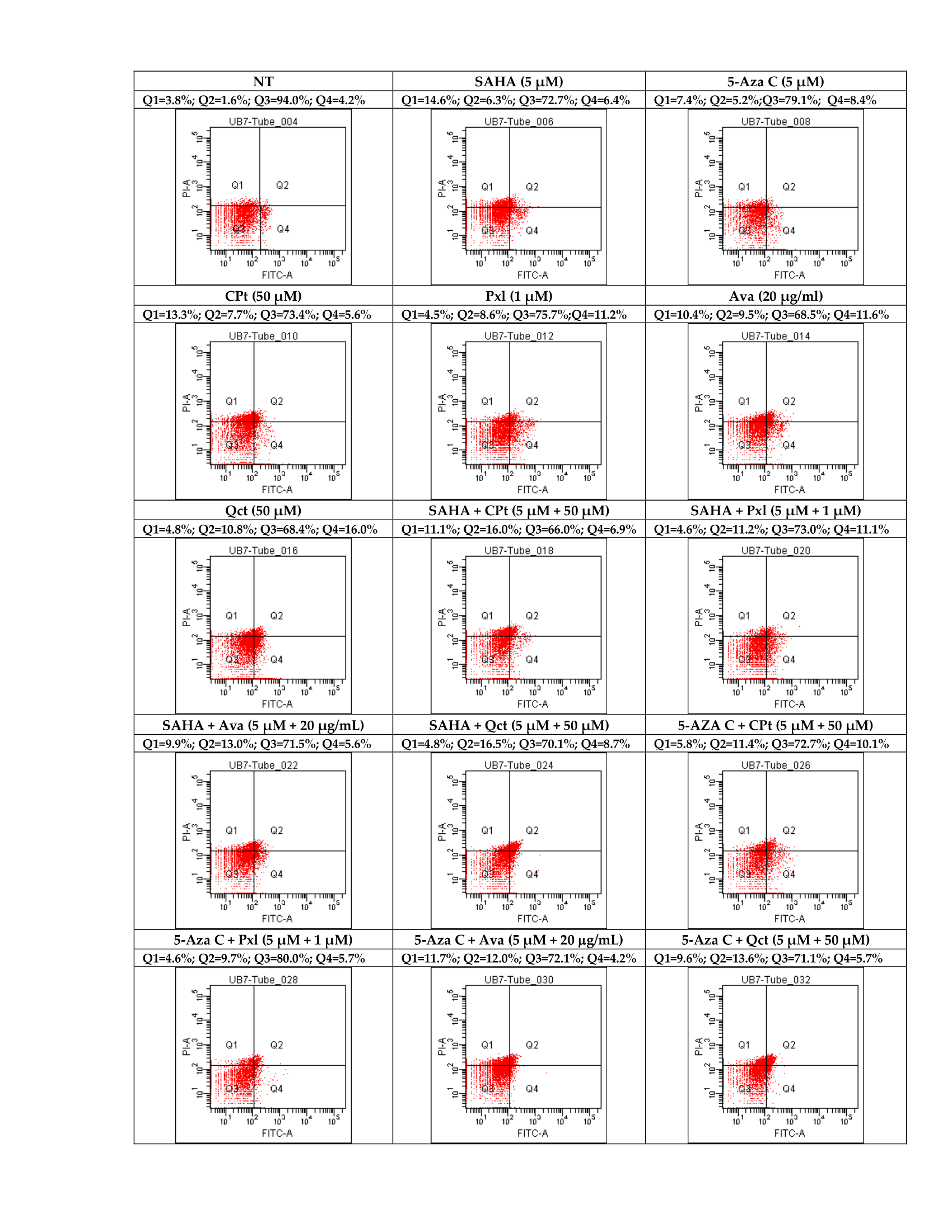

Supplement: Supplementary file 1 [file genes-16-00138-s001.zip › Fig S2.tiff]
